# Effect of Snyder's hope theory-based nursing intervention on patients with breast cancer

**DOI:** 10.1590/1980-220X-REEUSP-2024-0305en

**Published:** 2025-07-28

**Authors:** Yiling Jing, Huali Hu

**Affiliations:** 1 Yongkang First People’s Hospital, Second Department of Surgery, Yongkang, Zhejiang Province, China.

**Keywords:** Breast Neoplasms, Drug Therapy, Psychosocial Intervention, Nursing, Neoplasias da Mama, Tratamento Farmacológico, Intervencao Psicossocial, Enfermagem

## Abstract

**Objective:**

We aimed to assess the effect of Snyder’s Hope Theory-based nursing intervention on patients with breast cancer receiving postoperative chemotherapy.

**Method:**

The subjects (110 in total) were recruited between June 2023 and May 2024 and assigned into a control group (n = 55) and an intervention group (n = 55) using random numbers. For the control group, routine intervention was conducted, while for the intervention group, Snyder’s Hope Theory-based nursing intervention was implemented. The duration of interventions in both groups was 4 weeks.

**Results:**

After intervention, the scores of Self-rating Anxiety Scale and Self-rating Depression Scale declined markedly in the intervention group in comparison with those in the control group (P < 0.05). Compared with the control group, the intervention group had obviously higher total scores of Herth Hope Index and Functional Assessment of Cancer Therapy-Breast scales, as well as scores of all dimensions therein (P < 0.05).

**Conclusion:**

Snyder’s Hope Theory-based nursing intervention can effectively alleviate the negative emotions of anxiety and depression, enhance the hope level, and mitigate cancer-related fatigue.

## RESUMO

**Objetivo:**

Avaliar o efeito da intervenção de enfermagem baseada na Teoria da Esperança de Snyder em pacientes com câncer de mama submetidas à quimioterapia pós-operatória.

**Método:**

Os participantes (110 no total) foram recrutados entre junho de 2023 e maio de 2024 e divididos em grupo controle (n = 55) e grupo intervenção (n = 55), utilizando números aleatórios. O grupo controle recebeu intervenção rotineira, enquanto o grupo intervenção recebeu a intervenção de enfermagem baseada na Teoria da Esperança de Snyder. A duração das intervenções em ambos os grupos foi de 4 semanas.

**Resultados:**

Após a intervenção, as pontuações das escalas de Ansiedade e Depressão (EAS e EDS) diminuíram significativamente no grupo intervenção em comparação com o grupo controle (P < 0,05). Em relação ao grupo controle, o grupo intervenção apresentou pontuações totais significativamente mais altas no Índice de Esperança de Herth e na Escala de Avaliação Funcional do Câncer de Mama (FACT-B), bem como em todas as dimensões dessas escalas (P < 0,05).

**Conclusão:**

A intervenção de enfermagem baseada na Teoria da Esperança de Snyder pode reduzir efetivamente emoções negativas como ansiedade e depressão, aumentar o nível de esperança e mitigar a fadiga relacionada ao câncer.

## INTRODUCTION

As a common postoperative adjuvant therapy for breast cancer, chemotherapy is able to suppress the progression and prevent the recurrence of lesions, but it inevitably leads to relatively more toxic and adverse reactions because chemotherapeutic drugs work by specifically inhibiting or killing proliferating and active cancer cells in the body. In addition, most of the patient’s physical strength and energy is consumed by the disease. This results in anxiety, depression and other negative emotions^(1,2)^. If such negative emotions are not effectively relieved for a long time, patients will suffer from cancer- related fatigue, which is a feeling of fatigue that interferes with the normal physiological function of the human body^(3,4)^. Cancer-related fatigue is difficult to manage and predict, and exercise, health education, and drug therapy have limited effectiveness on it, so it is of great importance to discover effective non-pharmacological interventions. Routine nursing has been used in the past for breast cancer patients undergoing postoperative chemotherapy and has facilitated the recovery of physical function in these patients to some extent, but the overall effect is limited. The level of hope not only has a significant correlation with the physical and mental health of patients, but also has an important effect on cancer-related fatigue^(5)^. There is a negative relationship between cancer-related fatigue and hope level^(6)^. Based on this, increasing the hope level may be a possible way to alleviate cancer-related fatigue in breast cancer patients undergoing postoperative chemotherapy in clinical practice. Snyder’s Hope Theory, a cognitive dynamic theory model, aims to help patients regain confidence and hope in treatment, which may have a positive significance in alleviating cancer-related fatigue in patients^(7)^. However, clinical studies on the effect of a nursing intervention based on Snyder’s Hope Theory on hope levels are lacking.

Therefore, in the present study, a nursing intervention based on Snyder’s Hope Theory was applied to breast cancer patients receiving postoperative chemotherapy to evaluate its effects on negative emotions, hope level, cancer-related fatigue, quality of life, compliance with nursing care, and satisfaction with nursing care.

## METHOD

### Subjects

In this prospective study, 110 patients with breast cancer who underwent postoperative chemotherapy in our hospital from June 2023 to May 2024 were enrolled and evenly divided into a control group (n = 55) and an intervention group (n = 55) using the random number method. Specifically, the enrolled patients were randomly assigned using a computer- generated randomization sequence. This sequence was generated by an independent statistician to ensure allocation concealment. The random numbers were generated in a 1:1 ratio to ensure equal distribution between the two groups. Sequentially numbered, opaque, sealed envelopes containing the group assignments were prepared and managed by a third party not involved in patient recruitment or data collection. Upon enrollment, each patient was assigned to a group by opening the next envelope in sequence, thereby maintaining allocation concealment and minimizing selection bias. General data including age, Karnofsky Performance Scale score, education level, surgical mode, tumor node metastasis stage, body mass index, Eastern Cooperative Oncology Group score, and monthly household income were comparable between the two groups (P > 0.05) (Table S1 in the Supplementary Appendix).

### Ethical Aspects Subsection

This study was ethically approved by the hospital and all patients signed the informed consent form (approval number YKSDYRMYYEC2023-KT-HS-010-01).

### Inclusion and Exclusion Criteria

The inclusion criteria were as follows: 1) patients who were clinically diagnosed with breast cancer and received chemotherapy for &gt;2 times, 2) those with an expected survival time of more than half a year, and 3) those with normal cognitive function, communication ability, and audiovisual perception.

Exclusion criteria included: 1) patients who were illiterate, 2) those with severe dysfunction of major organs, 3) those simultaneously undergoing other treatments, such as radiotherapy and targeted therapy, 4) those complicated with diseases of the blood system, immune system or endocrine system, 5) those complicated with uncontrolled communicable or infectious diseases, or 6) those unable to complete 4 cycles of chemotherapy or stay in the research due to various factors.

### Methods for Control Group (Routine Nursing Intervention)

**Figure 1:**
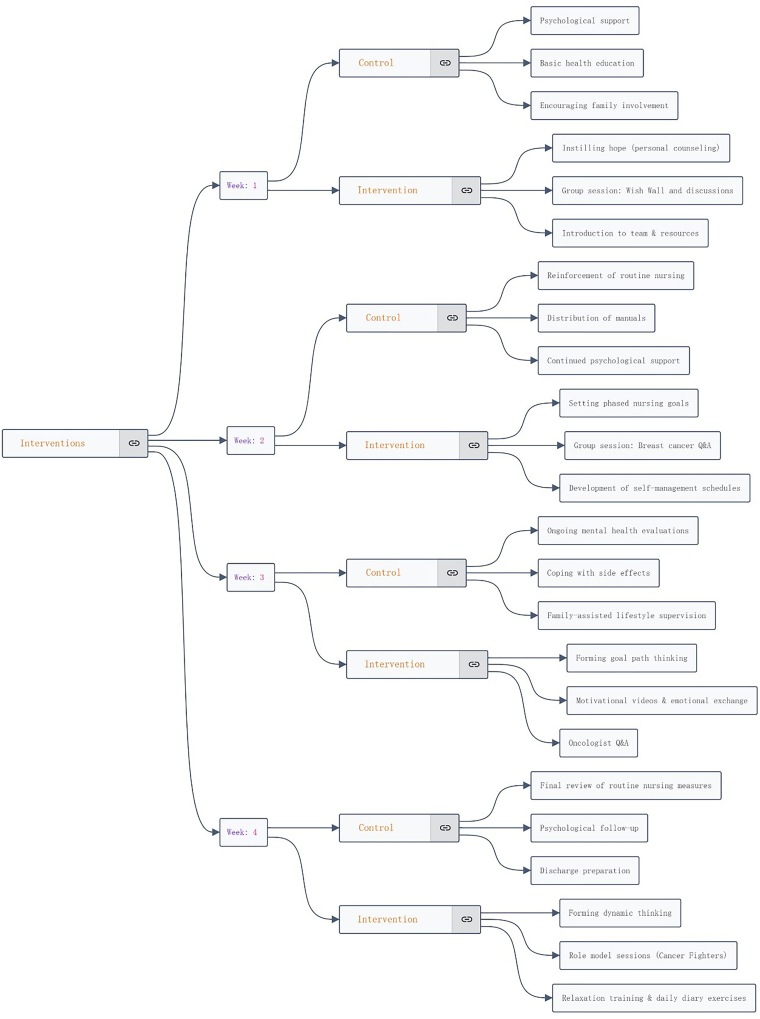
Intervention methods for two groups.

Patients in the control group received routine nursing interventions to address psychological well-being, provide health education, and guide lifestyle management (Figure 1).

(1) Psychological support was provided through regular communication before and after each chemotherapy session, during which patients’ mental health was assessed and tailored counseling was provided to address anxiety and depression. Family members were encouraged to spend time with patients to enhance emotional support. In addition, expert sessions were organized so that caregivers and patients could share knowledge and experiences regarding disease management and care strategies.
(2) Health education focused on disseminating information about chemotherapy procedures, potential side effects, and the importance of adherence. Educational materials, including handbooks and informational videos, were provided to reinforce key points about chemotherapy and cancer-related fatigue.
(3) Patients were further supported by a life-coaching component that included individualized nutrition and rest plans. They were guided to develop healthy lifestyle habits (such as moderate exercise and regular sleep patterns) and to involve family members in monitoring and encouraging these habits. These interventions were delivered face-to-face in inpatient or outpatient settings, depending on patient availability, and continued for four weeks.

### Methods for Intervention Group (Snyder’s Hope Theory-Based Nursing Intervention)

For the intervention group, a dedicated care team consisting of a primary care nurse, an attending physician, a nurse manager, and a psychological counselor implemented an intervention based on Snyder’s Hope Theory. This was delivered over four consecutive weeks, each with specific tasks designed to enhance hope, goal-setting skills, and pathways thinking (Figure 1).

(1) The first week focused on instilling hope. Nurses introduced team members, clarified the goals of the intervention, and created opportunities for patients to express and discuss their concerns. Interactive group sessions, such as activities around a “Wish Wall” and the “Chinese Pass the Parcel” game, were organized to encourage the sharing of positive messages and supportive experiences. These sessions typically lasted about 60 minutes and were designed to boost patient morale and strengthen social connections among participants.
(2) During the second week, the care team worked with each patient to set incremental goals based on individual health conditions and personal preferences. These goals were clearly displayed so that patients could monitor their progress. Group intervention activities (lasting 30–45 minutes) focused on assessing patients’ knowledge of breast cancer through questionnaires, clarifying misconceptions in real time, and guiding patients to develop individualized self-management plans. Family involvement was encouraged to ensure ongoing support.
(3) The third week focused on the formation of goal-path thinking. Sessions (lasting 30–45 minutes) included discussions of techniques for managing negative emotions, sharing motivational cancer-fighting videos, and answering patients’ medical questions through direct interaction with oncologists. These discussions highlighted recent research findings to help patients reframe their perspective on breast cancer treatment and foster a renewed confidence in their ability to cope with the disease.
(4) In the final week, the program emphasized the development of dynamic thinking. The nursing team hosted a “Learning from Role Models to Gain Energy” session, inviting successful “cancer fighters” to share their insights on living with cancer. A subsequent session, “Regulating Mentality and Rebuilding Hope,” introduced relaxation training and provided supportive materials such as videos and positive messages through a hospital-approved communication platform. Participants were encouraged to record meaningful daily events in a journal to increase their sense of control and optimism. All components in the intervention group were delivered either through face-to-face contact or through online support channels (such as WeChat) when scheduling required remote communication. These sessions, which lasted approximately 30–45 minutes each, were scheduled once or twice a week, with self-guided relaxation and diary activities recommended daily. If patients showed signs of increased anxiety or decreased engagement, the frequency and content of these sessions were adjusted to meet individual needs.

### Evaluation of Negative Emotions

Patients’ depression and anxiety levels were assessed using the Self-rating Depression Scale (SDS) and the Self-rating Anxiety Scale (SAS) before intervention (at the time of admission) and after intervention (4 weeks after intervention), respectively^(8)^. The above scales consisted of 20 items, and a 5-point Likert scoring method (0–4 points) was used. The standard score was calculated according to the following equation: standard score = raw score of all items × 1.25, which ranged from 0 to 100 points and was inversely related to the degree of depression and anxiety.

### Evaluation of Hope Level

The Herth Hope Index (HHI) scale was used before and after the intervention^(9)^. The scale consisted of 12 items in 3 dimensions, namely, having a positive attitude toward reality and the future, taking positive actions, and maintaining close relationships with others, and a 4–point Likert scoring method (1–4 points) was used. The total score ranged from 12 points (the lowest) to 48 points (the highest), and a higher score indicated a higher level of hope.

### Evaluation of Cancer-Related Fatigue

Cancer-related fatigue was assessed using the Revised Piper Fatigue Scale (RPFS) before and after the intervention^(10)^. The scale contained 4 open-ended (unscored) questions and a total of 22 items in 4 dimensions (behavioral, emotional, somatic, and cognitive). The VAS scoring method (0–10 points) was used. The total score was 0–220 points, which was positively associated with the level of cancer-related fatigue.

### Evaluation of Quality of Life

Quality of life was assessed using the Functional Assessment of Cancer Therapy-Breast (FACT-B) scale before and after intervention^(11)^. The scale consisted of 36 items (18 negative and 18 positive) in five dimensions, namely social and family status, emotional status, functional status, physiological status, and additional concerns. A 5-point Likert scoring method (0–4 points) was used. Positive and negative item scores were calculated as follows: positive item score = (0 + number of response options), negative item score = (4-number of response options). The total score was 0–140 points, and it had a positive correlation with the level of quality of life.

### Evaluation of Compliance to Nursing

Nursing compliance was categorized as good compliance (strict adherence to physician advice, with a high degree of cooperation with nursing interventions), moderate compliance (strict adherence to physician advice, with occasional noncooperation with nursing interventions), and poor compliance (nonadherence to physician advice, with no cooperation with nursing interventions).

### Evaluation of Satisfaction with Nursing

Nursing satisfaction was assessed by a nursing satisfaction questionnaire designed by our hospital after the intervention (reliability: 0.85; validity: 0.90). The self-designed questionnaire consisted of 10 items in four dimensions (nursing skills, theoretical knowledge, nursing responsibility, and service attitude) with a total score of 100 points (90–100 points for very satisfied, 70–89 points for basically satisfied, 60–69 points for generally satisfied, and ≤60 points for dissatisfied). Satisfaction with care = (very satisfied + basically satisfied).

### Statistical Analysis

Data analysis and processing were performed using SPSS 22.0 software. The normality of the data was subjected to the Shapiro-Wilk test, all of which conformed to normal distribution. Measurement data were expressed as mean ± standard deviation ( $\bar{\text{x}}$ ± s) and subjected to the t-test, while count data were expressed as percentages and subjected to the *χ* ^2^ test. P < 0.05 indicated that the difference was statistically significant.

## RESULTS

### Negative Emotions

SAS and SDS scores were significantly lower in the intervention group than in the control group after the intervention (P < 0.05) (Table 1).

**Table 1:** SAS and SDS scores ( $\bar{\text{x}}$ ± s, point) – Yongkang, Zhejiang Province, China, 2023–2024.

		SAS score		SDS score	
Group	n	Before intervention	After intervention	Before intervention	After intervention
Control	55	65.72 ± 5.15	45.11 ± 5.11^a^	62.36 ± 5.31	45.25 ± 5.30^a^
Intervention	55	65.74 ± 5.17	41.99 ± 5.15^a^	62.39 ± 5.30	41.88 ± 5.28^a^
*t*		0.020	3.189	0.030	3.341
P		0.984	0.002	0.976	0.001

^a^P < 0.05 *vs*. before intervention in the same group.

### Hope Level

Before the intervention, no significant differences were found in the total score of the HHI scale and the scores of all its dimensions between the intervention and control groups (P > 0.05). After intervention, these scores increased significantly in the intervention group compared to the control group (P < 0.05) (Table 2).

**Table 2:** Total score of HHI scale and score of each dimension therein ( $\bar{\text{x}}$ ± s, point) – Yongkang, Zhejiang Province, China, 2023–2024.

		Having a positive attitude towards reality and future		Taking positive actions		Keeping close relationships with others		Total score	
Group	n	Before intervention	After intervention	Before intervention	After intervention	Before intervention	After intervention	Before intervention	After intervention
Control	55	8.12 ± 1.32	12.35 ± 1.55^a^	8.26 ± 1.32	12.37 ± 1.54^a^	8.25 ± 1.25	12.37 ± 1.56^a^	24.63 ± 1.85	37.09 ± 4.55
Intervention	55	8.14 ± 1.35	13.30 ± 1.56^a^	8.29 ± 1.35	13.32 ± 1.55^a^	8.28 ± 1.27	13.31 ± 1.54^a^	24.72 ± 1.83	39.93 ± 4.57
*t*		0.079	3.204	0.118	3.225	0.125	3.180	0.257	3.266
P		0.938	0.002	0.906	0.002	0.901	0.002	0.798	0.002

^a^P < 0.05 *vs*. before intervention in the same group.

### Cancer-Related Fatigue

The total score of the RPFS and the score of each dimension within it showed no significant differences between the intervention group and the control group before the intervention (P > 0.05), after the intervention, however, they decreased significantly in the intervention group in contrast to the control group. (P < 0.05) (Table 3).

**Table 3:** Total score of RPFS and score of each dimension therein ( $\bar{\text{x}}$ ± s, point) - Yongkang, Zhejiang Province, China, 2023–2024.

		Behavior		Emotion		Body		Cognition		Total score	
Group	n	Before intervention	After intervention	Before intervention	After intervention	Before intervention	After intervention	Before intervention	After intervention	Before intervention	After intervention
Control	55	45.21 ± 5.35	32.35 ± 4.60^a^	38.30 ± 4.30	29.37 ± 2.54^a^	39.25 ± 4.22	28.37 ± 3.59^a^	49.60 ± 5.81	35.09 ± 4.56	172.36 ± 8.56	126.18 ± 7.21^a^
Intervention	55	45.23 ± 5.37	30.30 ± 4.62^a^	38.29 ± 4.31	27.75 ± 2.58^a^	39.27 ± 4.25	26.38 ± 5.54^a^	49.72 ± 5.83	32.20 ± 4.57	172.51 ± 8.60	116.63 ± 7.00^a^
*t*		0.020	2.332	0.012	3.318	0.025	2.236	0.108	3.320	0.092	7.048
P		0.984	0.022	0.990	0.001	0.980	0.027	0.914	0.001	0.927	0.000

^a^P < 0.05 *vs*. before intervention in the same group.

### Quality of Life

There were no significant differences in the total score of the FACT-B scale and the score of each dimension within it between the intervention group and the control group before the intervention (P > 0.05). Compared to the control group, the intervention group had a significantly higher total score on the FACT-B scale and scores on each of its dimensions after the intervention (P < 0.05) (Table 4).

**Table 4:** Total score of FACT-B scale and score of each dimension therein ( $\bar{\text{x}}$ ± s, point) – Yongkang, Zhejiang Province, China, 2023–2024.

		Physiological condition		Social and family status		Functional status		Emotional state		Additional concern		Total score	
Group	n	Before inter­vention	After inter­vention	Before inter­vention	After inter­vention	Before inter­vention	After inter­vention	Before inter­vention	After inter­vention	Before inter­vention	After inter­vention	Before inter­vention	After inter­vention
Control	55	8.65 ± 1.83	18.60 ± 3.94^a^	13.22 ± 2.51	18.41 ± 3.96^a^	12.57 ± 2.28	19.26 ± 4.64^a^	12.82 ± 2.83	16.69 ± 4.96^a^	17.28 ± 4.42	24.55 ± 5.91^a^	64.54 ± 3.56	97.51 ± 5.65^a^
Intervention	55	8.47 ± 1.98	21.00 ± 3.75^a^	13.36 ± 2.38	21.01 ± 4.32^a^	12.49 ± 2.30	22.06 ± 4.20^a^	12.61 ± 2.76	19.63 ± 4.22^a^	17.40 ± 4.27	28.00 ± 5.13^a^	64.33 ± 3.60	111.70 ± 6.57^a^
*t*		0.495	3.272	0.300	3.290	0.183	3.318	0.394	3.348	0.145	3.269	0.308	12.145
P		0.622	0.001	0.765	0.001	0.855	0.001	0.694	0.001	0.885	0.001	0.759	0.000

^a^P < 0.05 *vs*. before intervention.

### Compliance to Nursing

Nursing compliance was better in the intervention group than in the control group (P < 0.05) (Table 5).

**Table 5:** Comparison of compliance to nursing between the two groups [n (%)] – Yongkang, Zhejiang Province, China, 2023–2024.

Group	n	Good compliance	Moderate compliance	Poor compliance	Total compliance rate
Control	55	30 (54.55)	19 (34.55)	6 (10.91)	49 (89.09)
Intervention	55	35 (63.64)	20 (36.36)	0 (0.00)	55 (100.00)
χ^2^					4.407
P					0.036

### Satisfaction with Nursing

Satisfaction with care was significantly better in the intervention group than in the control group (P < 0.05) (Table S2, supplementary material).

## DISCUSSION

The level of cancer-related fatigue in patients can be negatively predicted by the level of hope^(11)^. Therefore, increasing patients’ hope level by effective measures is of great clinical significance to improve and alleviate cancer-related fatigue, eliminate negative emotions, improve quality of life, and promote improvement of prognosis. Snyder’s hope theory has been continuously applied to solve the physical and psychological diseases of various groups with satisfactory application results^(12,13)^. In this study, this theory was applied to breast cancer patients receiving postoperative chemotherapy. After the intervention, the intervention group showed a significant decrease in the scores of SAS and SDS, as well as a significant increase in the total score of the HHI scale and the scores of all dimensions therein, compared with those of the control group, indicating that the intervention effectively alleviated the patients’ negative emotions and increased their hope levels. According to a study on the sense of hope of family caregivers of patients with chronic diseases^(14)^, with the existence of “hope”, patients in practical dilemma still believed in the existence of many possibilities in the future, so they courageously looked forward to a bright future. The theoretical model of “hope” proposed on this basis serves as the basis for the intervention practice of current hope theory. Duggleby et al.^(15)^ found that interventions based on hope theory alleviated patients’ sad psychological state and made them optimistic about practical dilemmas and take positive actions. In addition, Samavi et al.^(16)^ reported that group hope therapy not only helped bedridden women to alleviate their physical and psychological discomfort, but also aroused their positive emotions.

This is mainly due to the following factors. First, based on Snyder’s Hope Theory, stepwise nursing interventions are formulated with clear themes for interventions in each phase (namely, instilling hope in the first week, setting goals in the second week, and forming path thinking and dynamic thinking in the third and fourth weeks, respectively), which ensures the scientificity and effectiveness of the interventions^(17,18)^. Second, patients are effectively imbued with hope and other positive ideas through face-to-face personal intervention (such as self-introduction, information support, and psychological counseling) and face-to-face group intervention (such as wish wall, self-telling, and open-ended questions) in the first week. In the second week, face-to-face communication is used to set individualized, stepwise goals for patients and to guide patients toward self-management and independent implementation of appropriate interventions. In the third and fourth weeks, online communication in the doctor-patient WeChat group and face-to-face group intervention are adopted to help patients form scientific way thinking and dynamic thinking. Positive ideas such as hope are permeated throughout the nursing intervention, which effectively restores the hope level of patients. Third, the hope level as a positive psychological resource not only provides an inexhaustible motive force for individuals to achieve their life goals, but also improves patients’ ability to cope with diseases and their sense of powerlessness in solving difficulties in time, thus effectively alleviating anxiety, depression and other negative emotions in diseases and treatment^(19,20)^.

Moreover, the results of this study showed that after the intervention, the total score of the FACT-B scale and the score of each item therein significantly increased, whereas the total score of the RPFS and the score of each item therein significantly decreased in the intervention group compared with those in the control group. This indicates that the nursing intervention based on Snyder’s Hope Theory effectively alleviates cancer- related fatigue and improves the quality of life of breast cancer patients undergoing postoperative chemotherapy.

This is mainly due to the following facts. First, patients who receive nursing intervention based on Snyder’s Hope Theory have a higher hope level, and a high hope level can urge patients to face the disease positively and take positive actions to relieve the pain during chemotherapy, which is conducive to alleviating cancer-related fatigue^(21)^. After alleviating physical, emotional, or cognitive fatigue, patients will feel physically and mentally well, which will contribute to the gradual change of their negative coping styles. As a result, they will communicate more frequently with medical staff, patients, and family members, effectively gaining a pleasant physical and mental experience and thus improving their quality of life. Second, the face-to-face (individual/group) and WeChat (individual/group) nursing interventions based on Snyder’s Hope Theory can help patients regain treatment hope, set treatment goals, form scientific path thinking and dynamic thinking, which helps to effectively build a strong social support system for patients from doctors, wards and families, thereby achieving the goals of regulating mentality and restoring hope. All of the above are conducive to improving patients’ subjective feelings in physiological condition, social and family status, functional status, emotional state and other aspects, thereby improving their quality of life. Furthermore, the results of this study manifested that the nursing compliance and satisfaction in the intervention group were better than those in the control group, proving that the nursing intervention based on Snyder’s Hope Theory can effectively increase the nursing compliance and satisfaction in breast cancer patients undergoing postoperative chemotherapy. This is because a high level of hope, a high level of quality of life, and a low level of negative emotions and cancer-related fatigue are capable of rekindling patients’ hope in life and increasing their confidence in the treatment of diseases, thereby enabling them to actively participate in nursing intervention practice and consciously cooperate with nurses in the implementation of appropriate nursing intervention measures, which is conducive to improving their intervention and satisfaction with nursing care.

However, this study is limited by its relatively small sample size and single-center design, which may affect the generalizability of the findings. Future research should include larger, multicenter studies and examine the long-term effects of hope theory-based interventions on emotional well-being, quality of life, and treatment outcomes in diverse patient populations.

## CONCLUSION

In conclusion, for breast cancer patients undergoing postoperative chemotherapy, nursing intervention based on Snyder’s Hope Theory can effectively alleviate their negative emotions of anxiety and depression as well as cancer-related fatigue, improve their hope level, and promote the improvement of their compliance and satisfaction with nursing care.

## DATA AVAILABILITY

The full dataset supporting the findings of this study is available upon request to the corresponding author.

## SUPPLEMENTARY MATERIAL

The following online material is available for this article:

Table S1 – Baseline data of two groups – Yongkang, Zhejiang Province, China, 2023–2024.

Table S2 – Satisfaction with nursing [n (%)] – Yongkang, Zhejiang Province, China, 2023–2024.
