# Supplementary material for: Effect of Snyder's hope theory-based nursing intervention on patients with breast cancer
Source: Rev Esc Enferm USP. 2025 Jul 28;59:e20240305. doi: 10.1590/1980-220X-REEUSP-2024-0305en (PMC12309523; doi:10.1590/1980-220X-REEUSP-2024-0305en)
Supplement: Supplementary file 1 [file 1980-220X-reeusp-59-e20240305-sup01.pdf]

Rev Esc Enferm USP

https://doi.org/10.1590/1980-220X-REEUSP-2024-0305en

Supplementary Material to “Effect of Snyder's Hope Theory-based nursing intervention on patients with breast cancer”

Table S1 - Baseline data of two groups - Yongkang, Zhejiang Province, China, 2023-2024.

Baseline data		Control group (n=55)	Intervention group (n=55)	Statistical value	P
Age ($\bar{x} \pm s$, year)		45.24±5.26	45.08±5.41	t=0.157	0.875
Karnofsky Performance Scale score ($\bar{x} \pm s$, point)		75.28±2.55	75.24±2.57	t=0.082	0.935
Education level [n (%)]	High school and above	30 (54.55)	32 (58.18)	χ^2=0.148	0.701
	Primary and junior high schools	25 (45.45)	23 (41.82)		
Surgical mode [n (%)]	Breast conserving surgery	20 (36.36)	22 (40.00)	χ^2=0.154	0.695
	Modified radical operation	35 (63.64)	33 (60.00)		
Tumor-node-metastasis stage [n (%)]	Stage I	27 (49.09)	25 (45.45)	χ^2=0.146	0.703
	Stage II-III	28 (50.91)	30 (54.55)		
Body mass index ($\bar{x} \pm s$, kg/m^2)		22.10±0.25	22.12±0.26	t=0.411	0.682
Eastern Cooperative Oncology Group score ($\bar{x} \pm s$, point)		1.25±0.21	1.23±0.20	t=0.512	0.610
Monthly household income ($\bar{x} \pm s$, CNY)		6000.26±500.27	6100.55±510.30	t=1.041	0.300
