# Supplementary material for: Effect of Snyder's hope theory-based nursing intervention on patients with breast cancer
Source: Rev Esc Enferm USP. 2025 Jul 28;59:e20240305. doi: 10.1590/1980-220X-REEUSP-2024-0305en (PMC12309523; doi:10.1590/1980-220X-REEUSP-2024-0305en)
Supplement: Supplementary file 2 [file 1980-220X-reeusp-59-e20240305-sup02.pdf]

Rev Esc Enferm USP

https://doi.org/10.1590/1980-220X-REEUSP-2024-0305en

Supplementary Material to "Effect of Snyder's Hope Theory-based nursing intervention on patients with breast cancer"

Table S2 - Satisfaction with nursing [n (%)] - Yongkang, Zhejiang Province, China, 2023-2024.

Group	n	Very satisfied	Basically satisfied	Generally satisfied	Dissatisfied	Total
Control	55	20 (36.36)	20 (36.36)	7 (12.73)	8 (14.55)	47 (85.45)
Intervention	55	28 (50.91)	17 (30.91)	10 (18.18)	0 (0.00)	55 (100.00)
χ^2						6.605
P						0.010
