# Supplementary material for: Effect of Snyder's hope theory-based nursing intervention on patients with breast cancer
Source: Rev Esc Enferm USP. 2025 Jul 28;59:e20240305. doi: 10.1590/1980-220X-REEUSP-2024-0305en (PMC12309523; doi:10.1590/1980-220X-REEUSP-2024-0305en)
Supplement: Supplementary file 3 [file 1980-220X-reeusp-59-e20240305-sup03.pdf]

Rev Esc Enferm USP

https://doi.org/10.1590/1980-220X-REEUSP-2024-0305en

Material Suplementar para “Efecto de la intervención de enfermería basada en la Teoría de la Esperanza de Snyder en pacientes con cáncer de mama”

Tabla S1 - Datos de referencia de dos grupos: Yongkang, provincia de Zhejiang, China, 2023-2024.

Datos de referencia		Grupo de control (n=55)	de intervención (n=55)	Valor estadístico	P
Edad ($\bar{x} \pm s$, año)		45,24 ± 5,26	45,08 ± 5,41	t = 0,157	0.875
Puntuación de la escala de desempeño de Karnofsky ($\bar{x} \pm s$, punto)		75,28 ± 2,55	75,24 ± 2,57	t = 0,082	0.935
Nivel de educación [n (%)]	Escuela secundaria y superior	30 (54,55)	32 (58.18)	χ^2 = 0,148	0.701
	Escuelas primarias y secundarias	25 (45.45)	23 (41.82)		
Modo quirúrgico [n (%)]	Cirugía conservadora de mama	20 (36.36)	22 (40.00)	χ^2 = 0,154	0.695
	Operación radical modificada	35 (63.64)	33 (60.00)		
tumor -nódulo-metástasis [n (%)]	Etapa I	27 (49.09)	25 (45.45)	χ^2 = 0,146	0.703
	Etapa II-III	28 (50.91)	30 (54,55)		
Índice de masa corporal ($\bar{x} \pm s$, kg/m^2)		22,10 ± 0,25	22,12 ± 0,26	t = 0,411	0.682
Puntuación del Eastern Cooperative Oncology Group ($\bar{x} \pm s$, punto)		1,25 ± 0,21	1,23 ± 0,20	t = 0,512	0.610
Ingresos mensuales del hogar ($\bar{x} \pm s$, CNY)		6000,26 ± 500,27	6100,55 ± 510,30	t = 1.041	0.300
