# Supplementary material for: Effect of Snyder's hope theory-based nursing intervention on patients with breast cancer
Source: Rev Esc Enferm USP. 2025 Jul 28;59:e20240305. doi: 10.1590/1980-220X-REEUSP-2024-0305en (PMC12309523; doi:10.1590/1980-220X-REEUSP-2024-0305en)
Supplement: Translation

# Translation: Efecto de la intervención de enfermería basada en la Teoría de la Esperanza de Snyder en pacientes con cáncer de mama

**DOI:** 10.1590/1980-220X-REEUSP-2024-0305es

**Authors:** Yiling Jing, Huali Hu

**Affiliations:** 1 Yongkang First People’s Hospital, Second Department of Surgery, Yongkang, Zhejiang Province, China.

**Keywords:** Neoplasias de la Mama, Quimioterapia, Intervencion Psicosocial, Enfermeria

## Abstract

**Objetivo:**

Evaluar el efecto de la intervención de enfermería basada en la Teoría de la Esperanza de Snyder en pacientes con cáncer de mama sometidos a quimioterapia postoperatoria.

**Método:**

Los participantes (110 en total) fueron reclutados entre junio de 2023 y mayo de 2024 y se dividieron en grupo control (n = 55) y grupo intervención (n = 55), con números aleatorios. El grupo control recibió una intervención de rutina, mientras que el grupo intervención, una de enfermería basada en la Teoría de la Esperanza de Snyder. La duración de las intervenciones en ambos grupos fue de 4 semanas.

**Resultados:**

Después de la intervención, las puntuaciones de las escalas de Ansiedad y Depresión (EAS y EDS) disminuyeron significativamente en el grupo intervención con relación al grupo control (P < 0,05). En contraposición, el grupo intervención obtuvo puntuaciones totales significativamente más altas en el Índice de Esperanza de Herth y en la Escala de Evaluación Funcional del Cáncer de Mama (FACT-B), así como en todas las dimensiones de dichas escalas (P < 0,05).

**Conclusión:**

La intervención de enfermería basada en la Teoría de la Esperanza de Snyder puede reducir eficazmente emociones negativas como la ansiedad y la depresión, aumentar el nivel de esperanza y aminorar la fatiga concerniente al cáncer.

## INTRODUCCIÓN

Como tratamiento adyuvante postoperatorio habitual del cáncer de mama, la quimioterapia es capaz de suprimir la progresión y prevenir la recidiva de las lesiones, pero inevitablemente provoca reacciones relativamente más tóxicas y adversas porque los fármacos quimioterapéuticos actúan inhibiendo o matando específicamente las células cancerosas proliferantes y activas del organismo. Además, la enfermedad consume la mayor parte de la fuerza física y la energía del paciente. Esto provoca ansiedad, depresión y otras emociones negativas^(1,2)^. Si estas emociones negativas no se alivian eficazmente durante mucho tiempo, los pacientes padecerán fatiga relacionada con el cáncer, que es una sensación de cansancio que interfiere en la función fisiológica normal del cuerpo humano^(3,4)^. La fatiga relacionada con el cáncer es difícil de controlar y predecir, y el ejercicio, la educación sanitaria y la farmacoterapia tienen una eficacia limitada sobre ella, por lo que es de gran importancia descubrir intervenciones no farmacológicas eficaces. En el pasado, la enfermería rutinaria se ha utilizado en pacientes con cáncer de mama sometidas a quimioterapia postoperatoria y ha facilitado hasta cierto punto la recuperación de la función física en estas pacientes, pero el efecto global es limitado. El nivel de esperanza no sólo tiene una correlación significativa con la salud física y mental de los pacientes, sino que también tiene un efecto importante en la fatiga relacionada con el cáncer^(5)^. Existe una relación negativa entre la fatiga relacionada con el cáncer y el nivel de esperanza^(6)^. Basándose en esto, aumentar el nivel de esperanza puede ser una posible forma de aliviar la fatiga relacionada con el cáncer en pacientes con cáncer de mama sometidas a quimioterapia postoperatoria en la práctica clínica. La Teoría de la Esperanza de Snyder, un modelo de teoría dinámica cognitiva, pretende ayudar a los pacientes a recuperar la confianza y la esperanza en el tratamiento, lo que puede tener un significado positivo en el alivio de la fatiga relacionada con el cáncer en los pacientes^(7)^. Sin embargo, faltan estudios clínicos sobre el efecto de una intervención de enfermería basada en la Teoría de la Esperanza de Snyder sobre los niveles de esperanza.

Por lo tanto, en el presente estudio, se aplicó una intervención de enfermería basada en la Teoría de la Esperanza de Snyder a pacientes con cáncer de mama que recibían quimioterapia postoperatoria para evaluar sus efectos sobre las emociones negativas, el nivel de esperanza, la fatiga relacionada con el cáncer, la calidad de vida, el cumplimiento de los cuidados de enfermería y la satisfacción con los cuidados de enfermería.

## MÉTODO

### Sujetos

En este estudio prospectivo, se inscribieron 110 pacientes con cáncer de mama que se sometieron a quimioterapia postoperatoria en nuestro hospital entre junio de 2023 y mayo de 2024 y se dividieron uniformemente en un grupo de control (n = 55) y un grupo de intervención (n = 55) utilizando el método de números aleatorios. En concreto, los pacientes inscritos fueron asignados aleatoriamente mediante una secuencia de aleatorización generada por ordenador. Esta secuencia fue generada por un estadístico independiente para garantizar la ocultación de la asignación. Los números aleatorios se generaron en una proporción de 1:1 para garantizar una distribución equitativa entre los dos grupos. Una tercera persona no implicada en el reclutamiento de pacientes ni en la recogida de datos preparó y gestionó sobres cerrados, opacos y numerados secuencialmente que contenían las asignaciones de grupo. Tras la inscripción, cada paciente fue asignado a un grupo abriendo el siguiente sobre en secuencia, manteniendo así la ocultación de la asignación y minimizando el sesgo de selección. Los datos generales, incluidos la edad, la puntuación en la escala de rendimiento de Karnofsky, el nivel educativo, la modalidad quirúrgica, el estadio de metástasis en ganglios tumorales, el índice de masa corporal, la puntuación del Eastern Cooperative Oncology Group y los ingresos familiares mensuales fueron comparables entre los dos grupos (P > 0,05) (Tabla S1 del Apéndice suplementario).

### Subsección de Aspectos Éticos

Este estudio fue aprobado éticamente por el hospital y todas las pacientes firmaron el consentimiento informado (número de aprobación YKSDYRMYYEC2023-KT-HS-010-01).

### Criterios de Inclusión y Exclusión

Los criterios de inclusión fueron los siguientes 1) pacientes con diagnóstico clínico de cáncer de mama y que recibieron quimioterapia durante > 2 veces, 2) aquellas con un tiempo de supervivencia previsto de más de medio año, y 3) aquellas con función cognitiva, capacidad de comunicación y percepción audiovisual normales.

Los criterios de exclusión incluían: 1) pacientes analfabetos, 2) con disfunción grave de órganos principales, 3) sometidos simultáneamente a otros tratamientos, como radioterapia y terapia dirigida, 4) complicados con enfermedades del sistema sanguíneo, inmunitario o endocrino, 5) complicados con enfermedades transmisibles o infecciosas no controladas, o 6) incapaces de completar 4 ciclos de quimioterapia o de permanecer en la investigación debido a diversos factores.

### Métodos Para el Grupo de Control (Intervención Rutinaria de Enfermería)

Los pacientes del grupo de control recibieron intervenciones rutinarias de enfermería para abordar el bienestar psicológico, proporcionar educación sanitaria y orientar la gestión del estilo de vida (Figura 1).

(1) Se proporcionó apoyo psicológico mediante la comunicación periódica antes y después de cada sesión de quimioterapia, durante la cual se evaluó la salud mental de los pacientes y se proporcionó asesoramiento personalizado para abordar la ansiedad y la depresión. Se animó a los familiares a pasar tiempo con los pacientes para mejorar el apoyo emocional. Además, se organizaron sesiones de expertos para que cuidadores y pacientes pudieran compartir conocimientos y experiencias sobre la gestión de la enfermedad y las estrategias de atención.
(2) La educación sanitaria se centró en difundir información sobre los procedimientos de quimioterapia, los posibles efectos secundarios y la importancia del cumplimiento terapéutico. Se proporcionó material educativo, incluidos manuales y vídeos informativos, para reforzar los puntos clave sobre la quimioterapia y la fatiga relacionada con el cáncer.
(3) Los pacientes recibieron además el apoyo de un componente de life-coaching que incluía planes individualizados de nutrición y descanso. Se les orientó para que desarrollaran hábitos de vida saludables (como ejercicio moderado y patrones de sueño regulares) y para que implicaran a los familiares en el monitoreo y el fomento de estos hábitos. Estas intervenciones se llevaron a cabo de forma presencial en centros hospitalarios o ambulatorios, en función de la disponibilidad del paciente, y se prolongaron durante cuatro semanas.

**Figura 1:** Métodos de intervención para los dos grupos.

### Métodos Para el Grupo de Intervención (Intervención de Enfermería Basada en la Teoría de la Esperanza de Snyder)

En el grupo de intervención, un equipo de atención especializada formado por una enfermera de atención primaria, un médico, una enfermera gestora y un asesor psicológico llevó a cabo una intervención basada en la Teoría de la Esperanza de Snyder. La intervención se llevó a cabo durante cuatro semanas consecutivas, cada una de ellas con tareas específicas diseñadas para mejorar la esperanza, las habilidades para establecer objetivos y el pensamiento de caminos (Figura 1).

(1) La primera semana se centró en infundir esperanza. Las enfermeras presentaron a los miembros del equipo, aclararon los objetivos de la intervención y crearon oportunidades para que los pacientes expresaran y debatieran sus preocupaciones. Se organizaron sesiones de grupo interactivas, como actividades en torno a un «Muro de los deseos» y el juego «Chino pasa el paquete», para fomentar el intercambio de mensajes positivos y experiencias de apoyo. Estas sesiones solían durar unos 60 minutos y estaban diseñadas para levantar la moral de los pacientes y reforzar las conexiones sociales entre los participantes.
(2) Durante la segunda semana, el equipo asistencial trabajó con cada paciente para establecer objetivos graduales basados en las condiciones de salud individuales y las preferencias personales. Estos objetivos se expusieron claramente para que los pacientes pudieran monitorear su progreso. Las actividades de intervención en grupo (de 30 a 45 minutos de duración) se centraron en evaluar los conocimientos de las pacientes sobre el cáncer de mama mediante cuestionarios, aclarar conceptos erróneos en tiempo real y guiar a las pacientes para que desarrollaran planes de autocontrol individualizados. Se fomentó la participación de la familia para garantizar un apoyo continuo.
(3) La tercera semana se centró en la formación del pensamiento objetivo. Las sesiones (de 30–45 minutos de duración) incluían debates sobre técnicas para gestionar las emociones negativas, compartir vídeos motivadores sobre la lucha contra el cáncer y responder a las preguntas médicas de los pacientes mediante la interacción directa con los oncólogos. En estos debates se destacaron los resultados de investigaciones recientes para ayudar a las pacientes a replantear su perspectiva sobre el tratamiento del cáncer de mama y fomentar una confianza renovada en su capacidad para hacer frente a la enfermedad.
(4) En la última semana, el programa hizo hincapié en el desarrollo del pensamiento dinámico. El equipo de enfermería organizó una sesión titulada «Aprender de los modelos para ganar energía», a la que invitó a «luchadoras contra el cáncer» de éxito para que compartieran sus puntos de vista sobre la vida con el cáncer. Una sesión posterior, «Regular la mentalidad y recuperar la esperanza», introdujo el entrenamiento en relajación y proporcionó materiales de apoyo como vídeos y mensajes positivos a través de una plataforma de comunicación aprobada por el hospital. Se animó a los participantes a anotar los acontecimientos diarios significativos en un diario para aumentar su sensación de control y optimismo. Todos los componentes del grupo de intervención se impartieron mediante contacto cara a cara o a través de canales de apoyo en línea (como WeChat) cuando la programación requería comunicación a distancia. Estas sesiones, que duraban aproximadamente de 30 a 45 minutos cada una, se programaban una o dos veces por semana, y se recomendaban actividades de relajación autoguiadas y un diario diario. Si los pacientes mostraban signos de aumento de la ansiedad o disminución del compromiso, la frecuencia y el contenido de estas sesiones se ajustaban para satisfacer las necesidades individuales.

### Evaluación de las Emociones Negativas

Los niveles de depresión y ansiedad de los pacientes se evaluaron mediante la Escala de Autoevaluación de la Depresión (Self-rating Depression Scale, SDS) y la Escala de Autoevaluación de la Ansiedad (Self-rating Anxiety Scale, SAS) antes de la intervención (en el momento del ingreso) y después de la intervención (4 semanas después de la intervención), respectivamente^(8)^. Las escalas mencionadas constaban de 20 ítems, y se utilizó un método de puntuación Likert de 5 puntos (0–4 puntos). La puntuación estándar se calculó según la siguiente ecuación: puntuación estándar = puntuación bruta de todos los ítems ×1,25, que oscilaba entre 0 y 100 puntos y estaba inversamente relacionada con el grado de depresión y ansiedad.

### Evaluación del Nivel de Esperanza

Se utilizó la escala Índice de Esperanza de Herth (Herth Hope Index, HHI) antes y después de la intervención^(9)^. La escala constaba de 12 ítems en 3 dimensiones, a saber, tener una actitud positiva hacia la realidad y el futuro, emprender acciones positivas y mantener relaciones estrechas con los demás, y se utilizó un método de puntuación Likert de 4 puntos (1–4 puntos). La puntuación total oscilaba entre 12 puntos (la más baja) y 48 puntos (la más alta), y una puntuación más alta indicaba un mayor nivel de esperanza.

### Evaluación de la Fatiga Relacionada con el Cáncer

La fatiga relacionada con el cáncer se evaluó mediante la Escala Revisada de Fatiga de Piper (Revised Piper Fatigue Scale, RPFS) antes y después de la intervención^(10)^. La escala contenía 4 preguntas abiertas (sin puntuación) y un total de 22 ítems en 4 dimensiones (conductual, emocional, somática y cognitiva). Se utilizó el método de puntuación de Escala Visual Análoga, EVA (0–10 puntos). La puntuación total fue de 0–220 puntos, que se asoció positivamente con el nivel de fatiga relacionada con el cáncer.

### Evaluación de la Calidad de Vida

La calidad de vida se evaluó mediante la escala de Evaluación Funcional de la Terapia de Cáncer de Mama (Functional Assessment of Cancer Therapy-Breast, FACT-B) antes y después de la intervención^(11)^. La escala constaba de 36 ítems (18 negativos y 18 positivos) en cinco dimensiones, a saber, estado social y familiar, estado emocional, estado funcional, estado fisiológico y preocupaciones adicionales. Se utilizó un método de puntuación Likert de 5 puntos (0–4 puntos). Las puntuaciones positivas y negativas de los ítems se calcularon de la siguiente manera: puntuación positiva del ítem = (0 + número de opciones de respuesta), puntuación negativa del ítem = (4-número de opciones de respuesta). La puntuación total fue de 0–140 puntos, y tuvo una correlación positiva con el nivel de calidad de vida.

### Evaluación del Cumplimiento de Enfermería

El cumplimiento de la enfermería se clasificó en buen cumplimiento (adhesión estricta a los consejos del médico, con un alto grado de cooperación con las intervenciones de enfermería), cumplimiento moderado (adhesión estricta a los consejos del médico, con falta de cooperación ocasional con las intervenciones de enfermería) y mal cumplimiento (falta de adhesión a los consejos del médico, sin cooperación con las intervenciones de enfermería).

### Evaluación de la Satisfacción con la Enfermería

La satisfacción de enfermería se evaluó mediante un cuestionario de satisfacción de enfermería diseñado por nuestro hospital después de la intervención (fiabilidad: 0,85; validez: 0,90). El cuestionario autodiseñado constaba de 10 ítems en cuatro dimensiones (habilidades de enfermería, conocimientos teóricos, responsabilidad de enfermería y actitud de servicio) con una puntuación total de 100 puntos (90–100 puntos para muy satisfecho, 70–89 puntos para básicamente satisfecho, 60–69 puntos para generalmente satisfecho y ≤ 60 puntos para insatisfecho). Satisfacción con la atención = (muy satisfecho + básicamente satisfecho).

### Análisis Estadístico

El análisis y el procesamiento de los datos se realizaron con el programa SPSS 22.0. La normalidad de los datos se sometió a la prueba de Shapiro-Wilk, y todos ellos se ajustaron a una distribución normal. Los datos de medición se expresaron como media ± desviación estándar ( $\bar{\text{x}}$ ± s) y se sometieron a la prueba t, mientras que los datos de recuento se expresaron como porcentajes y se sometieron a la prueba *χ* ^2^. P < 0,05 indicó que la diferencia era estadísticamente significativa.

## RESULTADOS

### Emociones negativas

Las puntuaciones de SAS y SDS fueron significativamente menores en el grupo de intervención que en el grupo de control después de la intervención (P < 0,05) (Tabla 1).

**Tabla 1:** Puntuaciones SAS y SDS ( $\bar{\text{x}}$ ± s, punto) – Yongkang, provincia de Zhejiang, China, 2023–2024.

		escore SAS		escore SDS	
Grupo	n	Antes de la intervención	Después de la intervención	Antes de la intervención	Después de la intervención
Control	55	65.72 ± 5.15	45.11 ± 5.11^a^	62.36 ± 5.31	45.25 ± 5.30^a^
Intervención	55	65.74 ± 5.17	41.99 ± 5.15^a^	62.39 ± 5.30	41.88 ± 5.28^a^
*t*		0.020	3.189	0.030	3.341
P		0.984	0.002	0.976	0.001

^a^P < 0,05 frente a antes de la intervención en el mismo grupo.

### Nivel de Esperanza

Antes de la intervención, no se encontraron diferencias significativas en la puntuación total de la escala HHI ni en las puntuaciones de todas sus dimensiones entre los grupos de intervención y control (P > 0,05). Después de la intervención, estas puntuaciones aumentaron significativamente en el grupo de intervención en comparación con el grupo de control (P < 0,05) (Tabla 2).

**Tabla 2:** Escore total de la escala HHI y puntuación de cada una de sus dimensiones ( $\bar{\text{x}}$ ± s, punto) – Yongkang, provincia de Zhejiang, China, 2023–2024.

		Tener una actitud positiva ante la realidad y el futuro		Adoptar medidas positivas		Mantener relaciones estrechas con los demás		Escore total	
Grupo	n	Antes de la intervención	Después de la intervención	Antes de la intervención	Después de la intervención	Antes de la intervención	Después de la intervención	Antes de la intervención	Después de la intervención
Control	55	8.12 ± 1.32	12.35 ± 1.55^a^	8.26 ± 1.32	12.37 ± 1.54^a^	8.25 ± 1.25	12.37 ± 1.56^a^	24.63 ± 1.85	37.09 ± 4.55
Intervención	55	8.14 ± 1.35	13.30 ± 1.56^a^	8.29 ± 1.35	13.32 ± 1.55^a^	8.28 ± 1.27	13.31 ± 1.54^a^	24.72 ± 1.83	39.93 ± 4.57
*t*		0.079	3.204	0.118	3.225	0.125	3.180	0.257	3.266
P		0.938	0.002	0.906	0.002	0.901	0.002	0.798	0.002

^a^P < 0,05 frente a antes de la intervención en el mismo grupo.

### Fatiga Relacionada con el Cáncer

La puntuación total del RPFS y la puntuación de cada dimensión dentro del mismo no mostraron diferencias significativas entre el grupo de intervención y el grupo control antes de la intervención (P > 0,05), después de la intervención, sin embargo, disminuyeron significativamente en el grupo de intervención en contraste con el grupo control. (P < 0,05) (Tabla 3).

**Tabla 3:** Puntuación total del RPFS y puntuación de cada dimensión en el mismo ( $\bar{\text{x}}$ ± s, punto) – Yongkang, provincia de Zhejiang, China, 2023–2024.

		Conducta		Emoción		Cuerpo		Cognitivo		Escore total	
Grupo	n	Antes de la intervención	Después de la intervención	Antes de la intervención	Después de la intervención	Antes de la intervención	Después de la intervención	Antes de la intervención	Después de la inter­vención	Antes de la inter­vención	Después de la inter­vención
Control	55	45.21 ± 5.35	32.35 ± 4.60^a^	38.30 ± 4.30	29.37 ± 2.54^a^	39.25 ± 4.22	28.37 ± 3.59^a^	49.60 ± 5.81	35.09 ± 4.56	172.36 ± 8.56	126.18 ± 7.21^a^
Intervención	55	45.23 ± 5.37	30.30 ± 4.62^a^	38.29 ± 4.31	27.75 ± 2.58^a^	39.27 ± 4.25	26.38 ± 5.54^a^	49.72 ± 5.83	32.20 ± 4.57	172.51 ± 8.60	116.63 ± 7.00^a^
*t*		0.020	2.332	0.012	3.318	0.025	2.236	0.108	3.320	0.092	7.048
P		0.984	0.022	0.990	0.001	0.980	0.027	0.914	0.001	0.927	0.000

^a^P < 0,05 frente a antes de la intervención en el mismo grupo.

### Calidad de Vida

No hubo diferencias significativas en la puntuación total de la escala FACT-B ni en la puntuación de cada una de sus dimensiones entre el grupo de intervención y el grupo control antes de la intervención (P > 0,05). En comparación con el grupo de control, el grupo de intervención tuvo una puntuación total significativamente mayor en la escala FACT-B y puntuaciones en cada una de sus dimensiones después de la intervención (P < 0,05) (Tabla 4).

**Tabla 4:** Puntuación total de la escala FACT-B y puntuación de cada una de sus dimensiones ( $\bar{\text{x}}$ ± s, punto) – Yongkang, provincia de Zhejiang, China, 2023–2024.

		Condición fisiológica		Estatus social y familiar		Estatus funcional		Estado emocional		Preocupación adicional		Escore total	
Grupo	n	Antes de la inter­vención	Después de la inter­vención	Antes de la inter­vención	Después de la inter­vención	Antes de la inter­vención	Después de la inter­vención	Antes de la inter­vención	Después de la inter­vención	Antes de la inter­vención	Después de la inter­vención	Antes de la inter­vención	Después de la inter­vención
Control	55	8.65 ± 1.83	18.60 ± 3.94^a^	13.22 ± 2.51	18.41 ± 3.96^a^	12.57 ± 2.28	19.26 ± 4.64^a^	12.82 ± 2.83	16.69 ± 4.96^a^	17.28 ± 4.42	24.55 ± 5.91^a^	64.54 ± 3.56	97.51 ± 5.65^a^
Intervención	55	8.47 ± 1.98	21.00 ± 3.75^a^	13.36 ± 2.38	21.01 ± 4.32^a^	12.49 ± 2.30	22.06 ± 4.20^a^	12.61 ± 2.76	19.63 ± 4.22^a^	17.40 ± 4.27	28.00 ± 5.13^a^	64.33 ± 3.60	111.70 ± 6.57^a^
*t*		0.495	3.272	0.300	3.290	0.183	3.318	0.394	3.348	0.145	3.269	0.308	12.145
P		0.622	0.001	0.765	0.001	0.855	0.001	0.694	0.001	0.885	0.001	0.759	0.000

^a^P < 0,05 *frente* a antes de la intervención.

### Cumplimiento de Enfermería

El cumplimiento de la enfermería fue mejor en el grupo de intervención que en el grupo de control (P < 0,05) (Tabla 5).

**Tabla 5:** Comparación del cumplimiento terapéutico entre los dos grupos [n (%)] – Yongkang, provincia de Zhejiang, China, 2023–2024.

Grupo	n	Buen cumplimiento	Cumplimiento moderado	Bajo cumplimiento	Tasa total de cumplimiento
Control	55	30 (54.55)	19 (34.55)	6 (10.91)	49 (89.09)
Intervención	55	35 (63.64)	20 (36.36)	0 (0.00)	55 (100.00)
χ^2^					4.407
P					0.036

### Satisfacción con los Cuidados

La satisfacción con los cuidados fue significativamente mejor en el grupo de intervención que en el grupo control (P < 0,05) (Tabla S2, material suplementario).

## DISCUSIÓN

El nivel de fatiga relacionada con el cáncer en los pacientes puede predecirse negativamente por el nivel de esperanza^(11)^. Por lo tanto, aumentar el nivel de esperanza de los pacientes mediante medidas eficaces es de gran importancia clínica para mejorar y aliviar la fatiga relacionada con el cáncer, eliminar las emociones negativas, mejorar la calidad de vida y promover la mejora del pronóstico. La teoría de la esperanza de Snyder se ha aplicado continuamente para resolver las enfermedades físicas y psicológicas de diversos grupos con resultados de aplicación satisfactorios^(12,13)^. En este estudio, esta teoría se aplicó a pacientes con cáncer de mama que recibían quimioterapia postoperatoria. Tras la intervención, el grupo de intervención mostró una disminución significativa de las puntuaciones de SAS y SDS, así como un aumento significativo de la puntuación total de la escala HHI y de las puntuaciones de todas las dimensiones de la misma, en comparación con las del grupo de control, lo que indica que la intervención alivió eficazmente las emociones negativas de las pacientes y aumentó sus niveles de esperanza. Según un estudio sobre el sentido de la esperanza de los cuidadores familiares de pacientes con enfermedades crónicas^(14)^, con la existencia de la «esperanza», los pacientes en dilema práctico seguían creyendo en la existencia de muchas posibilidades en el futuro, por lo que esperaban con valentía un futuro brillante. El modelo teórico de la «esperanza» propuesto sobre esta base sirve de fundamento para la práctica de intervención de la teoría de la esperanza actual. Duggleby et al.^(15)^ descubrieron que las intervenciones basadas en la teoría de la esperanza aliviaban el triste estado psicológico de los pacientes y les hacían ser optimistas ante los dilemas prácticos y emprender acciones positivas. Además, Samavi et al.^(16)^ informaron de que la terapia de esperanza en grupo no sólo ayudaba a las mujeres encamadas a aliviar su malestar físico y psicológico, sino que también despertaba sus emociones positivas.

 Esto se debe principalmente a los siguientes factores. En primer lugar, basándose en la Teoría de la Esperanza de Snyder, se formulan intervenciones enfermeras escalonadas con temas claros para las intervenciones en cada fase (a saber, infundir esperanza en la primera semana, establecer objetivos en la segunda semana y formar el pensamiento de trayectoria y el pensamiento dinámico en la tercera y cuarta semanas, respectivamente), lo que garantiza la cientificidad y la eficacia de las intervenciones^(17,18)^. En segundo lugar, a los pacientes se les infunde esperanza y otras ideas positivas mediante intervenciones personales cara a cara (como la autopresentación, el apoyo informativo y el asesoramiento psicológico) e intervenciones grupales cara a cara (como el muro de los deseos, el autorrelato y las preguntas abiertas) en la primera semana. En la segunda semana, la comunicación cara a cara se utiliza para establecer objetivos individualizados y escalonados para los pacientes y para guiarlos hacia la autogestión y la aplicación independiente de las intervenciones adecuadas. En la tercera y cuarta semanas, la comunicación en línea en el grupo WeChat médico-paciente y la intervención en grupo cara a cara se adoptan para ayudar a los pacientes a formar un pensamiento científico y un pensamiento dinámico. Las ideas positivas como la esperanza se impregnan a lo largo de la intervención de enfermería, lo que restablece eficazmente el nivel de esperanza de los pacientes. En tercer lugar, el nivel de esperanza como recurso psicológico positivo no sólo proporciona una fuerza motriz inagotable para que los individuos alcancen sus objetivos vitales, sino que también mejora la capacidad de los pacientes para hacer frente a las enfermedades y su sensación de impotencia para resolver las dificultades a tiempo, lo que alivia eficazmente la ansiedad, la depresión y otras emociones negativas en las enfermedades y el tratamiento^(19,20)^. Además, los resultados de este estudio mostraron que, tras la intervención, la puntuación total de la escala FACT-B y la puntuación de cada ítem de la misma aumentaron significativamente, mientras que la puntuación total de la RPFS y la puntuación de cada ítem de la misma disminuyeron significativamente en el grupo de intervención en comparación con las del grupo de control. Esto indica que la intervención de enfermería basada en la Teoría de la Esperanza de Snyder alivia eficazmente la fatiga relacionada con el cáncer y mejora la calidad de vida de las pacientes con cáncer de mama sometidas a quimioterapia postoperatoria.

Esto se debe principalmente a los siguientes hechos. En primer lugar, las pacientes que reciben una intervención de enfermería basada en la Teoría de la Esperanza de Snyder tienen un mayor nivel de esperanza, y un alto nivel de esperanza puede instar a las pacientes a afrontar la enfermedad de forma positiva y a emprender acciones positivas para aliviar el dolor durante la quimioterapia, lo que favorece el alivio de la fatiga relacionada con el cáncer^(21)^. Tras aliviar la fatiga física, emocional o cognitiva, los pacientes se sentirán física y mentalmente bien, lo que contribuirá al cambio gradual de sus estilos de afrontamiento negativos. Como resultado, se comunicarán con más frecuencia con el personal médico, los pacientes y los familiares, lo que les proporcionará una experiencia física y mental agradable y mejorará su calidad de vida. En segundo lugar, las intervenciones de enfermería cara a cara (individual/grupo) y WeChat (individual/grupo) basadas en la Teoría de la Esperanza de Snyder pueden ayudar a los pacientes a recuperar la esperanza en el tratamiento, establecer objetivos de tratamiento, formar un pensamiento de camino científico y un pensamiento dinámico, lo que ayuda a construir eficazmente un fuerte sistema de apoyo social para los pacientes por parte de los médicos, los pabellones y las familias, logrando así los objetivos de regular la mentalidad y restaurar la esperanza. Todo ello contribuye a mejorar los sentimientos subjetivos de los pacientes en cuanto a estado fisiológico, situación social y familiar, estado funcional, estado emocional y otros aspectos, mejorando así su calidad de vida. Además, los resultados de este estudio manifestaron que el cumplimiento y la satisfacción de enfermería en el grupo de intervención fueron mejores que los del grupo de control, lo que demuestra que la intervención de enfermería basada en la Teoría de la Esperanza de Snyder puede aumentar eficazmente el cumplimiento y la satisfacción de enfermería en pacientes con cáncer de mama sometidas a quimioterapia postoperatoria. Esto se debe a que un alto nivel de esperanza, un alto nivel de calidad de vida y un bajo nivel de emociones negativas y fatiga relacionada con el cáncer son capaces de reavivar la esperanza de los pacientes en la vida y aumentar su confianza en el tratamiento de las enfermedades, lo que les permite participar activamente en la práctica de la intervención de enfermería y cooperar conscientemente con las enfermeras en la aplicación de medidas de intervención de enfermería adecuadas, lo que favorece la mejora de su intervención y la satisfacción con los cuidados de enfermería.

Sin embargo, este estudio está limitado por el tamaño relativamente pequeño de la muestra y el diseño unicéntrico, lo que puede afectar a la generalizabilidad de los resultados. Las investigaciones futuras deberían incluir estudios más amplios y multicéntricos y examinar los efectos a largo plazo de las intervenciones basadas en la teoría de la esperanza sobre el bienestar emocional, la calidad de vida y los resultados del tratamiento en diversas poblaciones de pacientes.

## CONCLUSIÓN

En conclusión, para las pacientes con cáncer de mama sometidas a quimioterapia postoperatoria, la intervención de enfermería basada en la Teoría de la Esperanza de Snyder puede aliviar eficazmente sus emociones negativas de ansiedad y depresión, así como la fatiga relacionada con el cáncer, mejorar su nivel de esperanza y promover la mejora de su cumplimiento y satisfacción con los cuidados de enfermería.

## DISPONIBILIDAD DE DATOS

El conjunto de datos completo que respalda los hallazgos de este estudio está disponible a solicitud del autor correspondiente.

## MATERIAL SUPLEMENTARIO

El siguiente material suplementario está disponible en línea:

Tabla S1 – Datos de referencia de dos grupos: Yongkang, provincia de Zhejiang, China, 2023–2024.

Tabla S2 – Satisfacción con la enfermería [n (%)] – Yongkang, provincia de Zhejiang, China, 2023–2024.
